# Supplementary material for: Coupled Feedback Loops Involving PAGE4, EMT and Notch Signaling Can Give Rise to Non-Genetic Heterogeneity in Prostate Cancer Cells
Source: Entropy (Basel). 2021 Feb 26;23(3):288. doi: 10.3390/e23030288 (PMC7996788; doi:10.3390/e23030288)
Supplement: Supplementary file 1 [file entropy-23-00288-s001.pdf]

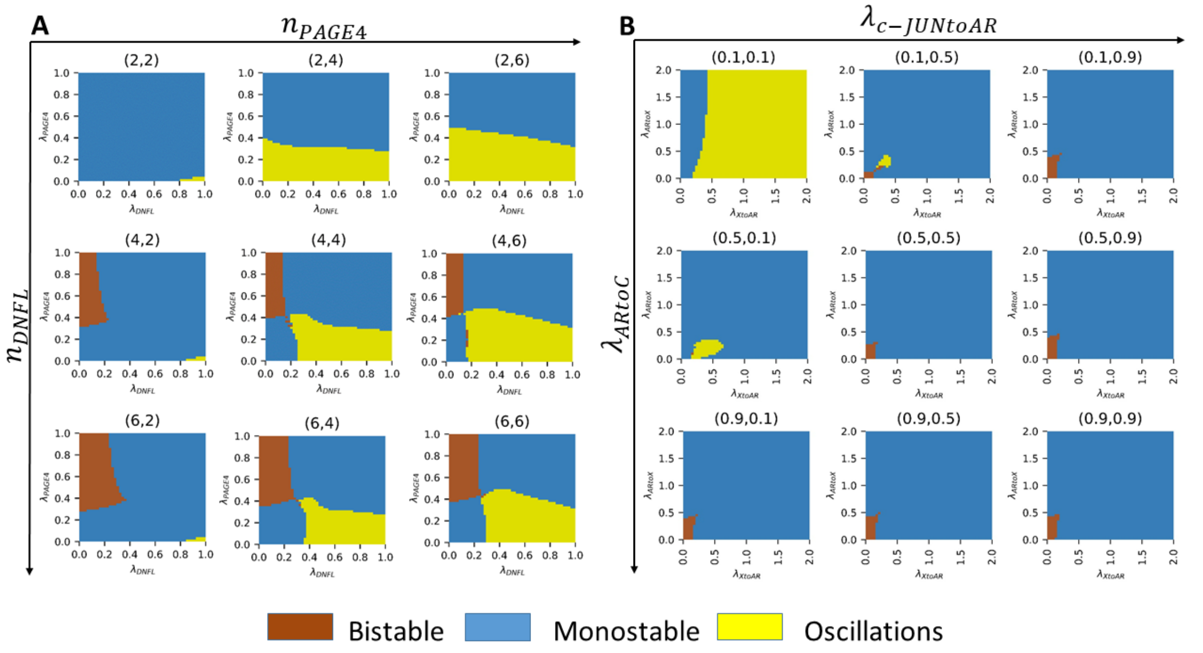

Figure S1: Sensitivity analysis A) *Effect of Hill-coefficients: Phase plot between λ_{DNFL} and λ_{PAGE4} for different values of Hill-coefficients for both these couplings. Yellow shading indicates oscillations, blue indicates monostability, and brown indicates bistability.* ***B)*** *Effect of different coupling strengths: Phase plot between λ_{AtoX} and λ_{ZtoA} for different strengths of internal coupling interactions (λ HIPK1-PAGE4 complex to AR (λ_{PMtoA}) and Lambda AR to CLK2 (λ_{AtoC})). For strong internal coupling (0.1, 0.1), the system shows oscillations even when X and A activate each other ($\lambda > 1$). But when the internal coupling is weak (0.9, 0.9), the system is largely monostable mainly and bistable in a small parameter region.*

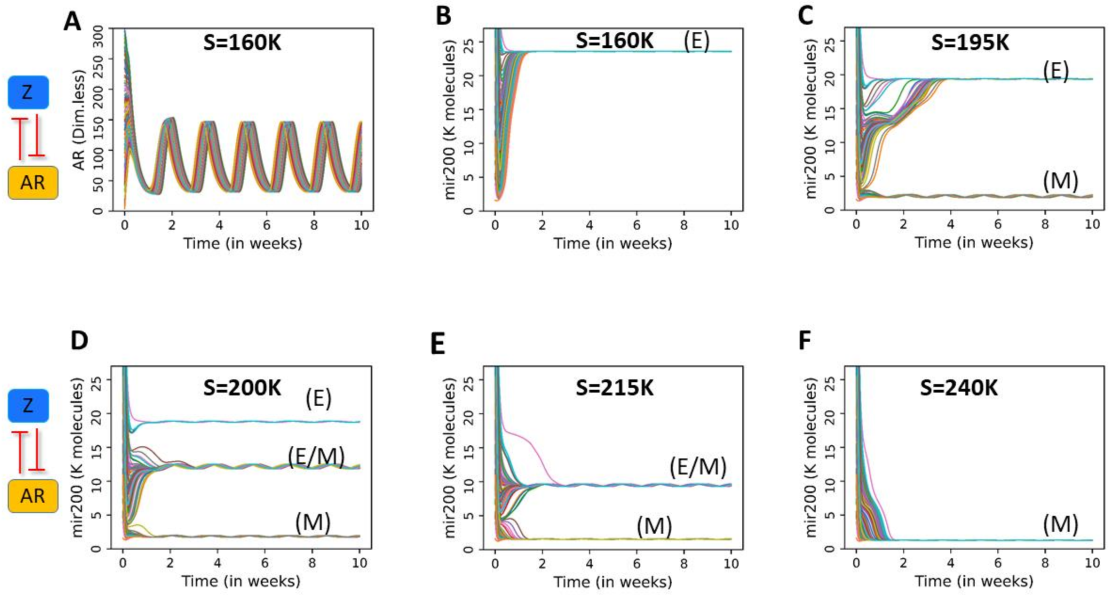

Figure S2: Dynamics at weak coupling between the EMT and PAGE4/AR circuits (λ_{AtoZ} and λ_{ZtoA} =0.9): A) *For strong internal coupling, AR shows oscillations.* ***B-F)*** *The EMT circuit shows small oscillations on top its steady states.*

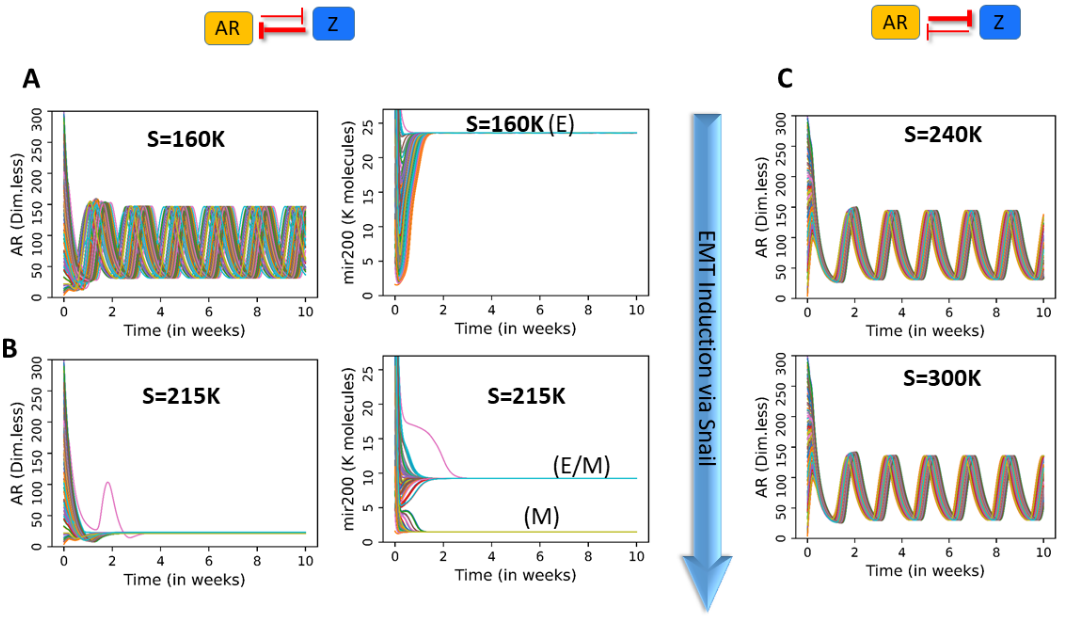

Figure S3: Dynamics of coupled circuits. A-B) *Dynamics of AR and miR-200 at varied levels of SNAIL as shown in panels, and at λ_{ZtoA}= 0.1 and λ_{AtoZ}= 0.9:* ***C)*** *AR dynamics at λ_{ZtoA}= 0.9 and λ_{AtoZ}= 0.1, for varied SNAIL levels.*

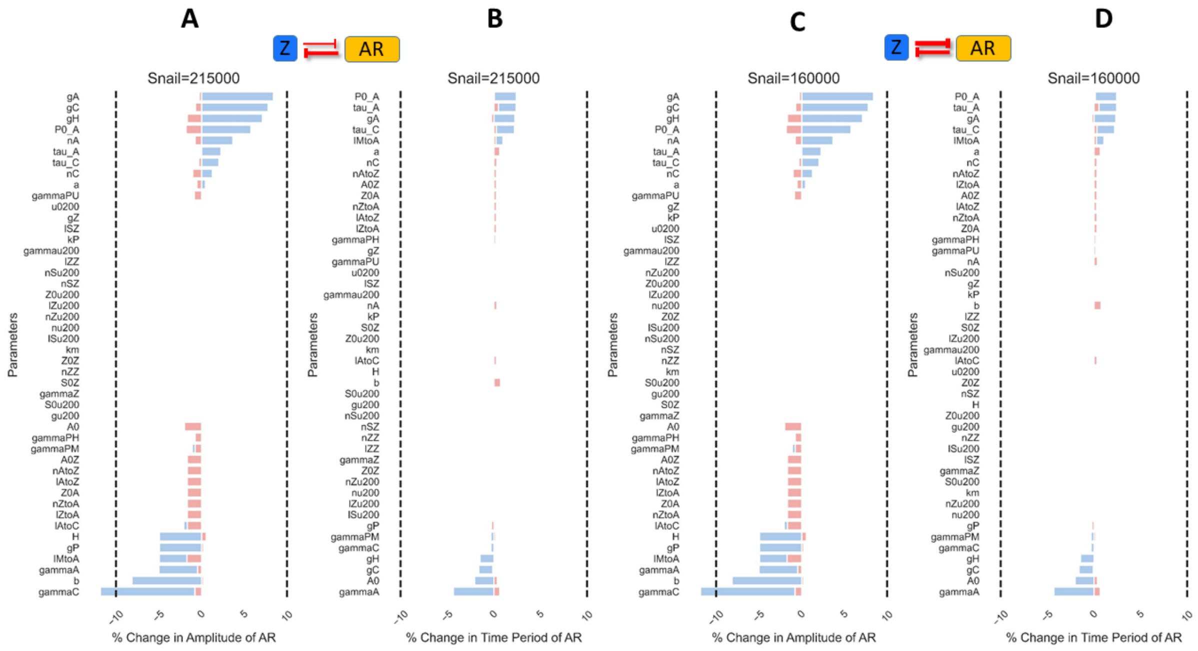

Figure S4: Sensitivity Analysis A-B) (λ_{ZtoA}=0.1 and λ_{AtoZ}=0.9): *Percentage change in Amplitude and Time period of AR for 10% variation in parameters. Snail levels are fixed at 215K here.* ***C-D) Same as A-B but for (λ_{ZtoA}= 0.1 and λ_{AtoZ}= 0.1):*** *Percentage change in Amplitude and Time period of AR for 10% variation in parameters. Snail levels are fixed 160K. For all panels, dotted lines correspond to -10% and +10% changes. Blue bars are for 10% increase in respective parameters whereas red bars are for 10% decrease in the same parameter.*

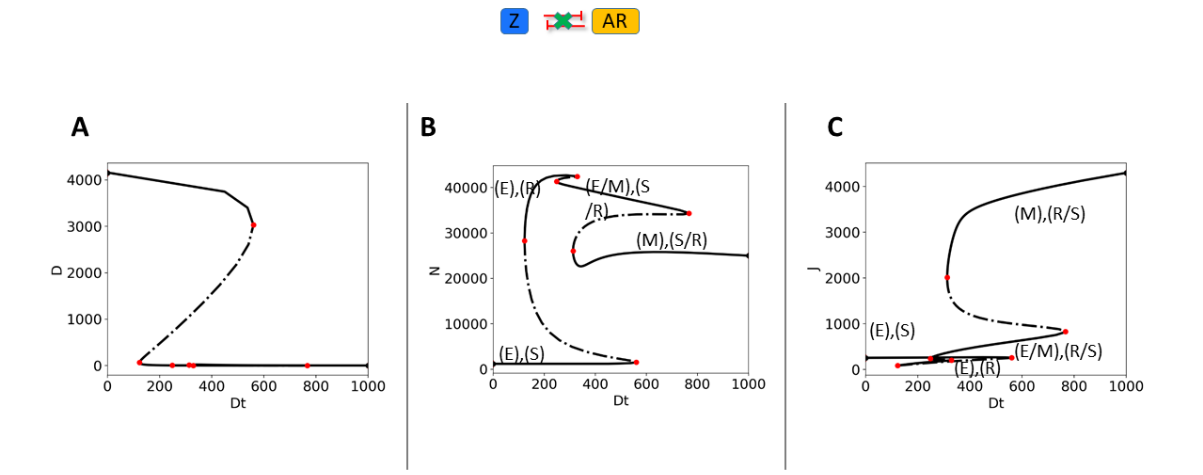

Figure S5: Bifurcation diagram of coupled EMT-Notch signaling. *Bifurcation plots of Delta (D), Notch (N) and Jagged (J) with respect to External Delta (Dext, referred to as Dt in above panel) for the case when PAGE4-AR and EMT circuits are not coupled. Dt is in number of molecules.*

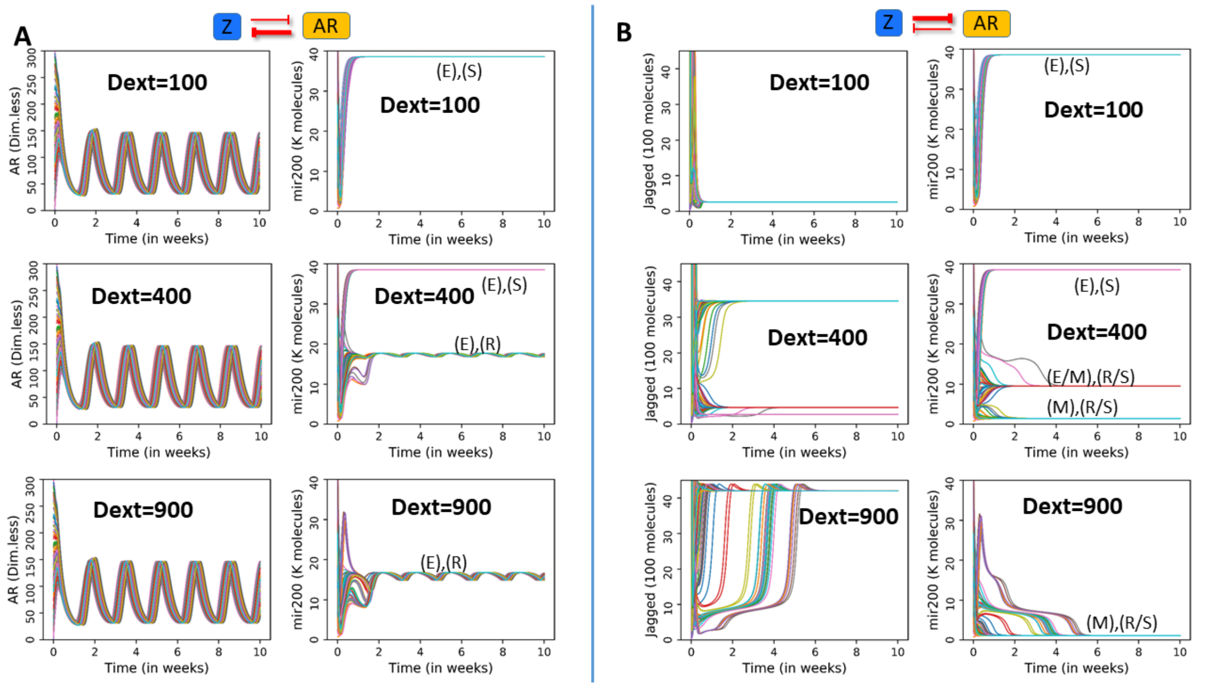

Figure S6: Dynamics of coupled circuits at varying coupling strengths. A) *Trajectories of AR and miR-200 at different Snail values for the case of* λ_{AtoZ}*=0.1 and* λ_{ZtoA}*=0.9. AR continues to oscillate. For low values of External Delta (top panel), miR-200 saturates at a value in the Epithelial Sender range. At higher external Delta (middle and lower panel) the epithelial receiver state appears and shows oscillations.* ***B)*** *Trajectories of Jagged and miR-200 at different Snail values for the case of* λ_{AtoZ}*=0.9 and* λ_{ZtoA}*=0.1. Jagged and miR-200 show similar behavior as to the uncoupled circuit (Fig 6B). Dext is in number of molecules.*

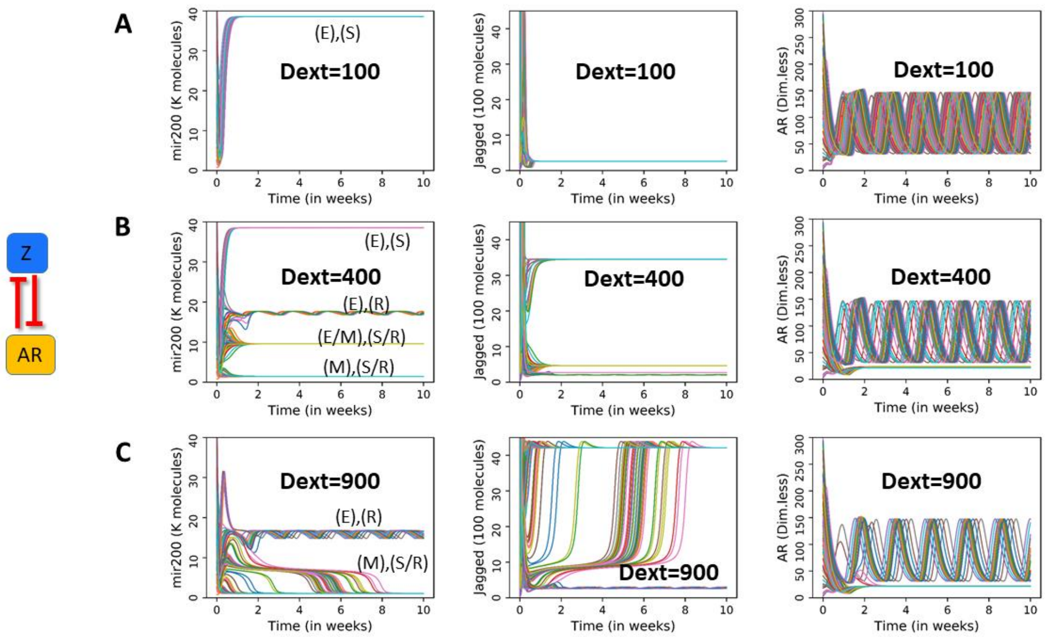

***Figure S7: PAGE4-EMT-NDJ circuit in case λ_{AtoZ} and λ_{ZtoA} =0.1: A)** On low external Delta, AR oscillates and EMT-NDJ circuit saturates at the epithelial sender state. At higher External Delta (Dext=400), cell choses either of the 4 possible states. The epithelial receiver state shows oscillations as well. At even higher External Delta (Dext=900), the hybrid and epithelial Sender state vanishes and only Epithelial Receiver and Mesenchymal (Sender/Receiver) state remain. Also, the epithelial receiver state continues to oscillates. AR either goes to an oscillatory trajectory or saturates at a low value. Dext is in number of molecules.*

Coupled feedback loops involving PAGE4,EMT and Notch signaling can give rise to non-genetic heterogeneity in prostate cancer cells

Supplementary Information

Divyoj Singh, Federico Bocci, Prakash Kulkarni, Mohit Kumar Jolly

1 PAGE4 - Androgen Receptor -X Module:

Building up from our previous work in [1], we first got correct dimensions of time. The absolute levels of the players are not known hence the levels are kept dimensionless. We also add another node “X” in the circuit to couple with AR.
Following are the equations for WT-PAGE4 (P_U), HIPK1-PAGE4 complex (P_M), CLK2-PAGE4 complex (P_H), CLK2 (C), Androgen Receptor (AR), new node ”X” (X) :

$$\dot{P_U}(t) = g_{P_U} - H \cdot \frac{P_u(t)}{P_u(t)+a} - \gamma_{P_u} P_u(t)$$

$$\dot{P_M}(t) = H\frac{P_u(t)}{P_u(t)+a} - g_H C(t)\frac{P_M(t)}{P_M(t)+b} - \gamma_{P_M} P_M(t)$$

$$\dot{P_H}(t) = g_H C(t)\frac{P_M(t)}{P_M(t)+b} - \gamma_{P_H} P_H(t)$$

$$\dot{C}(t) = g_C H^S(A(t-\tau_C), C) - \gamma_C C(t)$$

$$\dot{A}(t) = g_A H^S(X(t), A) H^S(P_M(t-\tau_A), A) - \gamma_A A(t)$$

$$\dot{X}(t) = g_X H^S(A(t), X) H^S(X(t), X) - \gamma_X X(t)$$

The terms $H^S(X(t), A)$ and $H^S(A(t), X)$ stand for the coupling terms of AR and X. The terms $H^S(P_M(t-\tau_A), A)$ and $H^S(A(t-\tau_C), C)$ stand for the delay terms due to the intermediate terms. All parameters are taken from [1] except for the parameters involving node ”X” which are estimated.

Table 1: Parameters for Page4-AR circuit

**Parameter**	**Value**	**Units**
H	2.31	$hour^{-1}$
Production rates:		
g_A	4.62	$hour^{-1}$
g_{P_U}	2.31	$hour^{-1}$
g_C	2.77	$hour^{-1}$
g_H	0.04	$hour^{-1}$
g_X	2.31	$hour^{-1}$
Degradation rates:		
γ_C	0.02	$hour^{-1}$
γ_{P_M}	0.004	$hour^{-1}$
γ_{P_H}	0.04	$hour^{-1}$
γ_{P_U}	0.016	$hour^{-1}$
γ_A	0.020	$hour^{-1}$
γ_X	0.04	$hour^{-1}$
Threshold constants:		
a	5	dimensionless
b	20	dimensionless
$P0A$	20	dimensionless
$A0$	65	dimensionless
$X0$	25	dimensionless

Hill Coefficient:		
n_A	4	dimensionless
n_C	4	dimensionless
n_X	4	dimensionless
Delay constants:		
τ_A	16.23	hour
τ_C	16.23	hour
Fold-change (regulation strength):		
λ_{AtoC}	0.1	dimensionless
λ_{MtoA}	0.1	dimensionless
λ_{XtoA}	1	dimensionless
λ_{AtoX}	1	dimensionless
λ_{XtoX}	1	dimensionless

2 PAGE4-AR-EMT Module:

To model EMT network,we first reduce the framework from [2] as done in [3]. Next, we replace X in the previous section with Zeb(Z). All other equations remain same. The parameters for EMT circuit are taken from [2] and [3].

$$\dot{P_U}(t) = g_{P_U} - H \cdot \frac{P_u(t)}{P_u(t) + a} - \gamma_{P_u} P_u(t)$$

$$\dot{P_M}(t) = H \frac{P_u(t)}{P_u(t) + a} - g_H C(t) \frac{P_M(t)}{P_M(t) + b} - \gamma_{P_M} P_M(t)$$

$$\dot{P_H}(t) = g_H C(t) \frac{P_M(t)}{P_M(t) + b} - \gamma_{P_H} P_H(t)$$

$$\dot{A}(t) = g_A \boldsymbol{H^S(Z(t), A)} H^S(P_M(t - \tau_A), A) - \gamma_A A(t)$$

$$\dot{C}(t) = g_C H^S(A(t - \tau_C), C) - \gamma_C C(t)$$

$$\dot{Z}(t) = k_P g_Z \boldsymbol{H^S(A(t), Z)} H^S(Z(t), Z) H^S(S(t), Z) Pl(\mu_{200}(t), 6) - \gamma_Z Z(t)$$

$$\dot{\mu_{200}}(t) = g_{\mu_{200}} H^S(S(t), \mu_{200}) H^S(Z(t), \mu_{200}) - g_Z H^S(Z(t), Z) \cdot H^S(S(t), Z) Py(\mu_{200}(t), 6) - \gamma_{\mu_{200}} \mu_{200}(t)$$

$$\dot{S}(t) = S(t)$$

Table 2: Parameters for core-EMT circuit

**Parameter**	**Value**	**Units**
Translation rate:		
k_P	1.0×10^2	proteins per mRNA per hour
Degradation rate of mRNA:		
k_m	5.0×10^{-1}	per hour
Production rates:		
$g_{\mu_{200}}$	2.1×10^3	molecules/hour
g_Z	11	molecules/hour
Degradation rate:		
$\gamma_{\mu_{200}}$	5.0×10^{-2}	$hour^{-1}$
γ_Z	1.0×10^{-1}	$hour^{-1}$
Threshold constants:		
$S0\mu_{200}$	1.8×10^5	molecules
$S0Z$	1.8×10^5	molecules
$Z0\mu_{200}$	2.2×10^5	molecules
$Z0Z$	2.2×10^5	molecules
$A0Z$	65	molecules
$Z0A$	2.5×10^4	molecules

Hill Coefficient:		
$n_{Zto\mu_{200}}$	3	dimensionless
$n_{Sto\mu_{200}}$	2	dimensionless
n_{StoZ}	2	dimensionless
n_{ZtoZ}	2	dimensionless
n_{ZtoA}	4	dimensionless
n_{AtoZ}	4	dimensionless
Fold-change (regulation strength):		
$\lambda_{Zto\mu_{200}}$	0.1	dimensionless
$\lambda_{Sto\mu_{200}}$	0.1	dimensionless
λ_{StoZ}	10	dimensionless
λ_{ZtoZ}	7.5	dimensionless
λ_{ZtoA}	0.1	dimensionless
λ_{AtoZ}	0.1	dimensionless

Table 3: Parameters for translation, mRNA degradation and micro-RNA degradation upon protein-micro-RNA binding

**Parameter group**	**Parameter**	**Value**	**Units**
Translation rate	l_i	1.0, 0.6, 0.3, 0.1, 0.05, 0.05, 0.05	h^{-1}
mRNA degradation rate	γ_{mi}	0, 0.04, 0.2, 1.0, 1.0, 1.0, 1.0	h^{-1}
micro-RNA degradation rate	$\gamma_{\mu i}$	0, 0.005, 0.05, 0.5, 0.5, 0.5, 0.5	h^{-1}

3 PAGE4-EMT-NDJ:

For combining the N-D-J circuitry, we add ODE's for Snail(S), microRNA-34(μ_{34}), Notch (N), Delta(D), Jagged (J) and NICD(I) from [3]. All parameters are taken from [3].

$$\dot{P_U}(t) = g_{P_U} - H \cdot \frac{P_u(t)}{P_u(t)+a} - \gamma_{P_u} P_u(t)$$

$$\dot{P_M}(t) = H\frac{P_u(t)}{P_u(t)+a} - g_H C(t)\frac{P_M(t)}{P_M(t)+b} - \gamma_{P_M} P_M(t)$$

$$\dot{P_H}(t) = g_H C(t)\frac{P_M(t)}{P_M(t)+b} - \gamma_{P_H} P_H(t)$$

$$\dot{C}(t) = g_C H^S(A(t-\tau_C), C) - \gamma_C C(t)$$

$$\dot{A}(t) = g_A H^S(Z(t), A) H^S(P_M(t-\tau_A), A) - \gamma_A A(t)$$

$$\dot{Z}(t) = k_P g_Z H^S(A(t), Z) H^S(Z(t), Z) H^S(S(t), Z) Pl(\mu_{200}(t), 6) - \gamma_Z Z(t)$$

$$\dot{\mu_{200}}(t) = g_{\mu_{200}} H^S(S(t), \mu_{200}) H^S(Z(t), \mu_{200}) - g_Z H^S(Z(t), Z) \cdot H^S(S(t), Z) Py(\mu_{200}(t), 6) - g_J H^S(I(t), \mu_{200}) Py(\mu_{200}(t), 5) - \gamma_{\mu_{200}} \mu_{200}(t)$$

$$\dot{S}(t) = k_P g_S H^S(S(t), S) H^S(I(t), S) H^S(It, S) Pl(\mu_{34}(t), 2) - \gamma_S S(t)$$

$$\dot{\mu_{34}}(t) = g_{\mu_{34}} H^S(S(t), \mu_{34}) H^S(Z(t), \mu_{34}) - g_S H^S(S(t), S) H^S(I(t), S) H^S(It, S) Py(\mu_{34}(t), 2) - g_N H^S(I(t), N) Py(\mu_{34}(t), 2) - g_D H^S(I(t), D) Py(\mu_{34}(t), 3) - \gamma_{\mu_{34}} \mu_{34}(t)$$

$$\dot{N}(t) = kPg_N H^S(I(t), N) Pl(\mu_{34}(t), 2) - N(t)((kcD(t) + ktDt) H^S(I(t), D) + (kcJ(t) + ktJt) H^S(I(t), J)) - \gamma_N N(t)$$

$$\dot{D}(t) = kPg_D H^S(I(t), D) Pl(\mu_{34}(t), 3) - D(t)(kcN(t) H^S(I(t), D) + ktNt) - \gamma_D D(t)$$

$$\dot{J}(t) = kPg_J H^S(I(t), J) Pl(\mu_{200}(t), 5) - J(t)(kcN(t) H^S(I(t), J) + ktNt) - \gamma_D D(t)$$

$$\dot{I}(t) = ktN(t)(DtH^S(I(t),D) + JtH^S(I(t),J)) - \gamma_I I(t)$$

Table 4: Parameters for EMT - NDJ circuit

**Parameter**	**Value**	**Units**
Production rates:		
$g_{\mu_{34}}$	1.35×10^3	molecules/hour
g_S	9×10^1	molecules/hour
g_N	0.8×10^1	molecules/hour
g_D	7×10^1	molecules/hour
g_J	2×10^1	molecules/hour
Degradation rates:		
$\gamma_{\mu_{34}}$	5.0×10^{-2}	$hour^{-1}$
γ_S	1.25×10^{-1}	$hour^{-1}$
γ_N	1.0×10^{-1}	$hour^{-1}$
γ_I	5.0×10^{-1}	$hour^{-1}$
γ_D	1.0×10^{-1}	$hour^{-1}$
γ_J	1.0×10^{-1}	$hour^{-1}$
Threshold constants:		
$S0\mu_{34}$	3×10^5	molecules
$S0S$	2×10^5	molecules
$Z0\mu_{34}$	2.2×10^5	molecules
$I0S$	3×10^2	molecules
$I0$	100	molecules
Hill Coefficient:		
$n_{Sto\mu_{34}}$	1	dimensionless
n_{StoS}	1	dimensionless
$n_{\mu_{34}}$	2	dimensionless
n_I	2	dimensionless
n_F	1	dimensionless
n_N, n_D, n_J	2	dimensionless
Fold-change (regulation strength):		
$\lambda_{Sto\mu_{200}}$	0.1	dimensionless
λ_{StoS}	0.1	dimensionless
$\lambda_{Zto\mu_{200}}$	0.2	dimensionless
λ_{ItoS}	6.5	dimensionless
λ_{ItoN}	7.0	dimensionless
λ_{ItoD}	0.0	dimensionless
λ_{ItoJ}	2.0	dimensionless
Cis-inhibition rate:		
kc	1.0×10^{-4}	$hour^{-1}$
Trans-activation rate:		
kt	1.0×10^{-5}	$hour^{-1}$
External Ligand and Notch Concentration:		
Dt	0.1	*molecules*
Nt	0.1	*molecules*
Jt	0.1	*molecules*
External Signal on Snail:		
It	0.0	*molecules*

4 Functions:

In our framework, we used following functions.

$$H^S(A(t), B(t)) = H^S(A, A0B, n_{AtoB}, \lambda_{AtoB}) = \frac{1}{1 + (\frac{A}{A0B})^{n_{AtoB}}} + \lambda \frac{(\frac{A}{A0B})^{n_{AtoB}}}{1 + (\frac{A}{A0B})^{n_{AtoB}}}$$

We discuss Shifted Hill function in detail in Materials and Methods in the main text.

The following functions appear in the EMT circuit which models effect of micro-RNA on gene expression. The formulation is formulated in detail in Supplementary Information of [2] and has been extended in [3].

$$P_l(\mu, n) = \frac{L(\mu, n)}{Y_m(\mu, n) + k_m}$$

This represents post-translational inhibition due to a micro-RNA species μ ($\mu 200 or \mu 34$ here). Here n represents the number of binding sites of micro-RNA on the promoter region of the target species. k_m represents the degradation rate of mRNA.

$$P_y(\mu, n) = \frac{Y_\mu(\mu, n)}{Y_m(\mu, n) + k_m}$$

represents the decrease in the levels of microRNA due to the degradation of the microRNA/mRNA complex.
Here, $L(\mu, n) = \sum_{i=0}^{n} l_i C_i^n M_i^n(\mu)$

$Y_m(\mu, n) = \sum_{i=0}^{n} \gamma_{mi} C_i^n M_i^n(\mu)$

$Y_m(\mu, n) = \sum_{i=0}^{n} \gamma_{\mu i} C_i^n M_i^n(\mu)$

$$C_i^n = \frac{n!}{i!(n-i)!}$$

and

$$M_i^n = \frac{(\frac{\mu}{\mu 0})^i}{(1 + \frac{\mu}{\mu 0})^n}$$

5 Phase plots for PAGE4 - AR -X Module:

The phase plot was divided into 50 x 50 cells. For each cell, at that parameter value, two trajectories were obtained, one starting with AR high and X low and other with AR low and X high. We used Euler-Integration with time step dt=0.001hr and for a total time of 10 weeks. After this, extrema of the second half of the trajectory were calculated, if the $\frac{difference\ of\ the\ extrema}{sum\ of\ the\ extrema} > 0.1$; then oscillatory

Otherwise, if: $\frac{differnce\ of\ steady\ state\ of\ trajectories}{sum\ of\ steady\ state\ of\ trajectories} > 0.1$; then bistable

Otherwise, it was considered monostable.
The phase plots were visualised using seaborn heatmap ([4]).

6 Dynamics Trajectories:

We first uniformly sample initial conditions from a specific range for each variable (say a random number between 0 and 300 for AR). Then we integrate the ODES's using Euler-Method with time step dt=0.001 hr and total duration of 10 weeks. The same process was repeated for 100 different trajectories for different initial conditions.

7 Phase plots for PAGE4-AR-EMT Module:

We first divided the phase space in 20 x 20 cells. Then for each cell, we calculated 100 trajectories, each starting with different initial conditions. The time step was fixed at dt=0.1hr and the trajectory was calculated for 100 weeks. Next we took the later half of the trajectory and calculated the amplitude (difference between the extrema) and the average value of both miR-200 and AR.
For AR:

$\frac{amplitude\ of\ AR}{average\ of\ AR} > 0.2$; then oscillatory

Otherwise, it was considered monostable.
Then we calculated average amplitude, accounting for only those cases out of total 100 cases where oscillations were observed according to the aforementioned condition.
For miR-200:

$average\ \mu_{200} > 15000$; then Epithelial

$15000 > average\ \mu_{200} > 5000$; then Epithelial Mesenchymal (Hybrid)

$5000 > average\ \mu_{200}$ then Mesenchymal

Now we calculated the fraction of initial conditions which go into each of the state and if this fraction is more than 2%, we consider that state to be present.

8 Sensitivity Analysis:

To perform the Sensitivity analysis, we first fix the value of $\lambda_{AtoC}, \lambda_{MtoA}, \lambda_{AtoZ}, \lambda_{ZtoA}$ *and S*. After this, we vary each of the parameters (one at a time) by +10% and -10%. We calculate the extrema of the later half of the trajectory and also calculate the time points at which these extrema occur. We thus calculate the variation in time period and amplitude of AR. These variations are plotted in Figure S4.

9 Bifurcation Diagrams:

The bifurcation diagram for miR-200 in Figure 1B was made in MATLAB using the package MATCONT [5] and was diagram is adapted from our previous study [2]. The bifurcation diagram for miR-200 in Figure 5B and Figure S4 were made in Python2 using PyDsTool ([6]).Figure 5B was also adapted from our previous study [3].

10 Codes:

These codes were written in Python3 and only basic libraries like numpy ([7]), scipy ([8]) and pandas ([9]) were used . The trajectories were visualised using matplotlib ([10]). All codes are available publicly on the GitHub page (https://github.com/Divyoj-Singh/PAGE4-AR-EMT-NDJ).

References

[1] X. Lin, S. Roy, M. K. Jolly, F. Bocci, N. P. Schafer, M. Y. Tsai, Y. Chen, Y. He, A. Grishaev, K. Weninger, J. Orban, P. Kulkarni, G. Rangarajan, H. Levine, and J. N. Onuchic, "PAGE4 and Conformational Switching: Insights from Molecular Dynamics Simulations and Implications for Prostate Cancer," *Journal of Molecular Biology*, vol. 430, no. 16, pp. 2422–2438, 2018, ISSN: 10898638. DOI: 10.1016/j.jmb.2018.05.011.

[2] M. Lu, M. K. Jolly, H. Levine, J. N. Onuchic, and E. Ben-Jacob, "MicroRNA-based regulation of epithelial-hybrid-mesenchymal fate determination," *Proceedings of the National Academy of Sciences of the United States of America*, vol. 110, no. 45, pp. 18 144–18 149, 2013, ISSN: 00278424. DOI: 10.1073/pnas.1318192110.

[3] M. Boareto, M. K. Jolly, A. Goldman, M. Pietilä, S. A. Mani, S. Sengupta, E. Ben-Jacob, H. Levine, and J. N. Onuchic, "Notch-Jagged signalling can give rise to clusters of cells exhibiting a hybrid epithelial/mesenchymal phenotype," *Journal of The Royal Society Interface*, vol. 13, no. 118, 2016, ISSN: 1742-5689. DOI: 10.1098/rsif.2015.1106.

[4] M. Waskom and the seaborn development team, *Mwaskom/seaborn*, version latest, Sep. 2020. DOI: 10.5281/zenodo.592845. [Online]. Available: https://doi.org/10.5281/zenodo.592845.

[5] A. Dhooge, W. Govaerts, and Y. A. Kuznetsov, "MATCONT: A MATLAB package for numerical bifurcation analysis of ODEs," *ACM Transactions on Mathematical Software*, vol. 29, no. 2, pp. 141–164, 2003, ISSN: 00983500. DOI: 10.1145/779359.779362. [Online]. Available: https://dl.acm.org/doi/10.1145/779359.779362.

[6] G. J. Clewley RH, Sherwood WE, LaMar MD, *PyDSTool, a software environment for dynamical systems modeling.* 2007. [Online]. Available: http://pydstool.sourceforge.net.

[7] C. R. Harris, K. J. Millman, S. J. van der Walt, R. Gommers, P. Virtanen, D. Cournapeau, E. Wieser, J. Taylor, S. Berg, N. J. Smith, R. Kern, M. Picus, S. Hoyer, M. H. van Kerkwijk, M. Brett, A. Haldane, J. F. del Río, M. Wiebe, P. Peterson, P. Gérard-Marchant, K. Sheppard, T. Reddy, W. Weckesser, H. Abbasi, C. Gohlke, and T. E. Oliphant, "Array programming with NumPy," *Nature*, vol. 585, no. 7825, pp. 357–362, 2020, ISSN: 0028-0836. DOI: 10.1038/s41586-020-2649-2. [Online]. Available: http://www.nature.com/articles/s41586-020-2649-2.

[8] P. Virtanen, R. Gommers, T. E. Oliphant, M. Haberland, T. Reddy, D. Cournapeau, E. Burovski, P. Peterson, W. Weckesser, J. Bright, S. J. van der Walt, M. Brett, J. Wilson, K. J. Millman, N. Mayorov, A. R. J. Nelson, E. Jones, R. Kern, E. Larson, C. J. Carey, l. Polat, Y. Feng, E. W. Moore, J. VanderPlas, D. Laxalde, J. Perktold, R. Cimrman, I. Henriksen, E. A. Quintero, C. R. Harris, A. M. Archibald, A. H. Ribeiro, F. Pedregosa, and P. van Mulbregt, "SciPy 1.0: fundamental algorithms for scientific computing in Python," *Nature Methods*, vol. 17, no. 3, 2020, ISSN: 1548-7091. DOI: 10.1038/s41592-019-0686-2.

[9] W. McKinney, "Data Structures for Statistical Computing in Python," in *Proceedings of the 9th Python in Science Conference*, SciPy, 2010, pp. 56–61. DOI: 10.25080/majora-92bf1922-00a.

[10] J. D. Hunter, "Matplotlib: A 2D graphics environment," *Computing in Science and Engineering*, vol. 9, no. 3, pp. 90–95, 2007, ISSN: 15219615. DOI: 10.1109/MCSE.2007.55.
